# The Vapors of Nitrate of Potassa in Asthma

**Published:** 1841-08

**Authors:** 


### THE VAPORS OF NITRATE OF POTASSA IN ASTHMA.

I have lately met with some cases of asthma, in which great relief was derived from inhaling the vapors arising from the decomposition of nitrate of potassa. The patients, after saturating white paper with a solution of the nitrate, and drying it thoroughly, set it on fire, and, dropping it into some close vessel, inhale the gases evolved by the combustion. A teapot answers well for the purpose, but it is sufficient to drop the ignited paper in a common glass tumbler, applying the mouth to it while it is filled with the vapors. The relief has been manifest in several cases, and in one, complete. The subject, a gentlemen aged 55 years, had been afflicted with asthma for more than twenty years, the paroxysms of which were marked with all the distress that attends that disease. For five years past he has been exempt from it, and his restoration he attributes entirely to this remedy. He was in the habit of carrying with him, in his pocketbook, paper prepared for the occasion, and of resorting to the fumes whenever he was threatened with an attack.

A lady, of about the same age, has derived great benefit from these inhalations, in the same disease. The paroxysm is always shortened, and greatly mitigated, by a resort to them.

At present, I have a patient under my charge, laboring under a pulmonary affection, one of the most afflicting symptoms in which is dyspnœa. For this, he has been inhaling the vapors of the nitrate for some days, and the result is, that he expectorates with more freedom and ease, and his breathing is much improved. In his case the remedy does not promise so much, as there is reason to fear the existence of organic lesions. Y.

